# Linking genotype, ecotype, and phenotype in an intensively managed large carnivore

**DOI:** 10.1111/eva.12122

**Published:** 2013-12-04

**Authors:** Aaron B A Shafer, Scott E Nielsen, Joseph M Northrup, Gordon B Stenhouse

**Affiliations:** 1 Department of Evolutionary Biology, Evolutionary Biology Centre, Uppsala UniversitetUppsala, Sweden; 2 Department of Renewable Resources, University of AlbertaEdmonton, AB, Canada; 3 Department of Fish, Wildlife and Conservation Biology, Colorado State UniversityFort Collins, CO, USA; 4 Fish and Wildlife Division, Foothills Research Institute and Alberta Environment and Sustainable Resource DevelopmentHinton, AB, Canada

**Keywords:** cluster analysis, genetics, global positioning system, grizzly bear, habitat use

## Abstract

Numerous factors influence fitness of free-ranging animals, yet often these are uncharacterized. We integrated GPS habitat use data and genetic profiling to determine their influence on fitness proxies (mass, length, and body condition) in a threatened population of grizzly bears (*Ursus arctos*) in Alberta, Canada. We detected distinct genetic and habitat use (ecotype) clusters, with individual cluster assignments, or genotype/ecotype, being correlated (Pearson *r *=* *0.34, *P *<* *0.01). Related individuals showed evidence of similar habitat use patterns, irrespective of geographic distance and sex. Fitness proxies were influenced by sex, age, and habitat use, and homozygosity had a positive effect on these proxies that could be indicative of outbreeding depression. We further documented over 300 translocations occurring in the province since the 1970s, often to areas with significantly different habitat. We argue this could be unintentionally causing the pattern of outbreeding, although the heterozygosity correlation may instead be explained by the energetic costs associated with larger body size. The observed patterns, together with the unprecedented human-mediated migrations, make understanding the link between genotype, ecotype, and phenotype and mechanisms behind the negative heterozygosity-fitness correlations critical for management and conservation of this species.

## Introduction

Finding a correlation between genetics and fitness-related traits in free-ranging vertebrates is challenging at best. Heterozygosity-fitness correlations (HFCs) have proven ambiguous (Chapman et al. 2009), while effective mapping approaches require pedigrees (Kruuk and Hadfield 2007). Still, attempting to characterize the links between genetic diversity and fitness remains an important pursuit, particularly in the context of wildlife conservation (Reed and Frankham 2003). These correlations are perhaps most relevant for the management of threatened and isolated populations, where inbreeding and the loss of genetic diversity can be mitigated through management actions such as human-mediated movement of conspecifics (Frankham et al. 2011).

Individual variation in habitat use and access to resources can also influence fitness-related traits in wild populations (Calsbeek 2009; Smith et al. 2010; Hoye et al. 2012). Competition for resources and subsequent natural selection has been suggested to initiate the development of ecomorphs (Calsbeek 2009), which when linked to genetic substructure is reflective of local adaptation or ecotypes (Turesson 1922). Ecological divergence can readily promote reproductive barriers and population differentiation (Nosil 2007), and importantly, if the observed ecomorphs are indeed due to adaptive divergence, a genetic signature should be present (Nosil 2012). Ecologically mediated genetic differentiation appears readily detectable within most wild species (Shafer and Wolf 2013), implying that species showing both ecological and genetic structuring are prime candidates for studying local adaptation. Current technology (i.e., GPS radiocollars) provides unprecedented access into the habitat use patterns of wild animals and has recently been coupled with genetic data to address questions related to gene flow and behavior (Shafer et al. 2012; Nielsen et al. 2013a). Integrating habitat use and genetic data into a fitness context is an important step as it allows inference on the relative contribution of each factor to the fitness of free-ranging individuals and goes beyond mere descriptive analyses. Identifying these links is particularly relevant for intensively managed species, as disrupting locally adapted alleles via outbreeding could have significant consequences (e.g., Atlantic salmon – McGinnity et al. 2003).

A relationship between heterozygosity and fitness reflects the spectrum from outbreeding to inbreeding depression. For close to three decades, empirical work has detected positive correlations between multilocus heterozygosity and phenotypes of fitness relevance (e.g., growth rate – Mitton and Grant 1984; parasite load – Coltman et al.^1999^; survival – Markert et al. 2004). Multilocus HFCs are thought to reflect genome-wide patterns of inbreeding, or the ‘general effects’ hypothesis (Hansson and Westerberg 2002), while if single locus is driving the relationship, typically it is due to so-called direct or local effects. As inbreeding depression has been well documented in the wild (Keller and Waller 2002), it is commonly invoked as the explanation for positive HFCs (see examples in Chapman et al. 2009). Outbreeding depression, alternatively, appears much less common (Edmands 2007) but is an important consideration, particularly when deciding whether to augment populations (Templeton 1986; Thornhill 1993; Frankham et al. 2011). Here, outbreeding depression arises from migrants breaking up co-adapted alleles or introducing nonadaptive genes that produce intermediate phenotypes (Lynch 1991; Edmands 1999; Szulkin and David 2011). It has also been experimentally shown that the crossing of genotypes adapted to differing environments can negatively impact growth (Tymchuk et al. 2007). Although Marshall and Spalton (2000) suggested outbreeding depression might be more common than initially perceived, negative HFCs have been only sparsely documented in the wild (e.g., DiBattista et al. 2008; Olano-Marin et al. 2011; Jourdan-Pineau et al. 2012; Monceau et al. 2012),

North America's grizzly bear (*Ursus arctos*) is an interesting species for studying genetic and ecological relationships to phenotypes and testing for local adaptation. Genetic data suggest large subpopulations, but with fine-scale differentiation often corresponding to landscape features (Proctor et al. 2011). There are distinct ecological groupings that can be made according to dietary (Mowat and Heard 2006), environmental, and life-history characteristics (Ferguson and McLoughlin 2008). Body size is a strong indicator of reproductive success in this species (Stringham 1990), but this trait varies considerably across the range (Nowak 1999) and is influenced by meat availability along coastal North America (Hilderbrand et al. 1999; McLellan 2011) and in some areas by density (Zedrosser et al. 2006).

In Alberta, Canada, the interior grizzly bear population of approximately 700 individuals (Festa-Bianchet and Kansas 2010) represents the southeastern periphery of the extant range of the species. Genetic substructure has formed in southern Alberta (Proctor et al. 2005), and provincewide five genetic clusters have been suggested (Proctor and Paetkau 2004), though considerable variation and overlap among clusters is evident (Proctor et al. 2011). Ecologically, Alberta bears are often classified as being either montane/foothill or alpine based on their primary use of habitat (Munro et al. 2006; Coogan et al. 2012). Grizzly bear diets in Alberta vary considerably (Robichaud 2009), and no effect of population density on body size has been detected (Nielsen et al. 2013bb). The Alberta grizzly bear population has recently been designated as threatened (Festa-Bianchet and Kansas 2010), a polarizing decision that has managers attempting to balance conservation, economic, and recreational interests (Chamberlain et al. 2012). As a hunting moratorium was issued in 2006, an average of 15 bears per year have died from human-related causes (Government of Alberta 2013). Further, issues related to livestock have continued to result in human–bear interactions and conflicts, often resulting in the capture and translocation of bears (Northrup and Boyce 2012). The intensive management of the species, including relatively high rates of mortality and translocation in some areas, has the potential to influence both phenotypic and genetic parameters of the population.

In this study, we examined the relationship among habitat use, genetic diversity and differentiation and three morphological traits considered to be fitness proxies (body condition, mass, and body length). Using genetic and GPS data from individual grizzly bears monitored since 1999, we tested for distinct genetic and habitat use clusters (that we refer to as ecotypes), and whether genetic relatedness was correlated with ecological distance after accounting for the confounding effects of geography: both of these can be viewed as signatures of local adaptation. In a mixed-model framework, we quantified the relationship among genetic diversity and differentiation, habitat use and the three fitness proxies. Finally, in an effort to shed some mechanistic insight into the purported patterns, we examined differences in habitat availability among management units and documented the translocation history of bears in the province using records dating back to the 1970s.

## Methods

### Database

**Figure 1:**
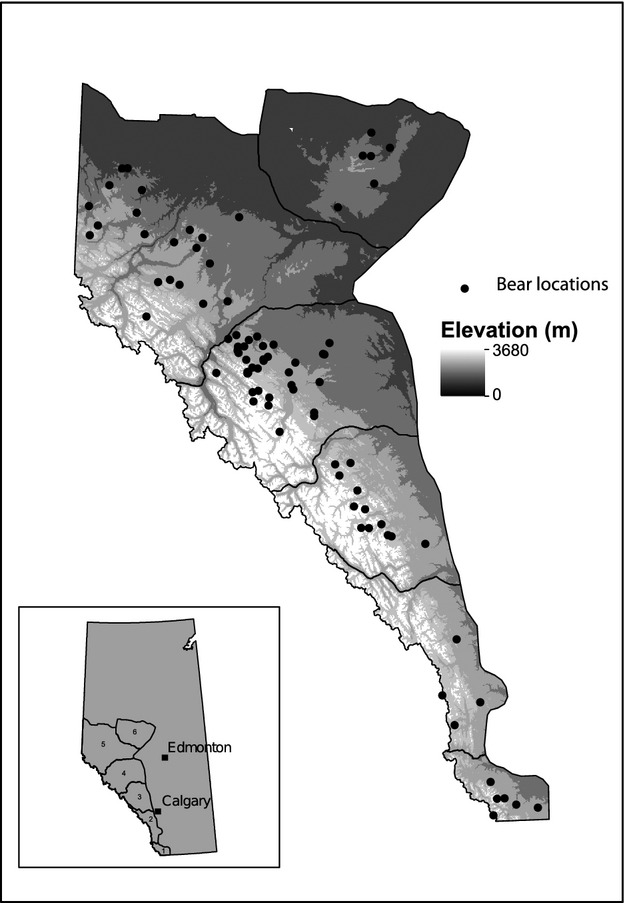
Map of grizzly bear (*Ursus arctos*) capture locations across the province of Alberta, Canada. Six management units are identified: (1) Castle, (2) Livingstone, (3) Clearwater, (4) Yellowhead, (5) Grande Cache, and 6) Swan Hills.

Our study area consisted of the eastern slope of the Canadian Rocky Mountains in Alberta, Canada and contained six of the seven provincially recognized grizzly bear management units (Fig. 1). Alberta is estimated to have 691 grizzly bears, of which 87% are found within our study area (Festa-Bianchet and Kansas 2010). Grizzly bears were captured between 1999–2008 using a combination of culvert traps, leg-hold snares, and heli-darting following the protocol of Cattet et al. (2003a,2003b). Each animal was fitted with either an Advanced Telemetry Systems (ATS) GPS radiocollar or Televilt GPS-Simplex radiocollar and were scheduled to attempt to acquire a location at 1-h and 4-h (pre 2003) intervals. Root hairs were collected from each bear for DNA analysis, and a premolar was extracted for aging (Stoneberg and Jonkel 1966). Each bear was weighed, measured for overall straight-line body length (SLL) from nose to tail, and a body condition index (BCI), which is a function of SLL and mass, was calculated following Cattet et al. (2002). If bears were captured multiple times, only the data from the first capture was used to avoid confounding effects of multiple captures (Cattet et al. 2008; Nielsen et al. 2013bb). All captures were approved by the University of Saskatchewan's Animal Care Committee and are in accordance with guidelines for handling of wildlife (CCAC 2003).

We measured individual-level use of seven land cover classes (wetland, cutblocks, shrubs, wetland herbaceous, upland herbaceous, barren, and upland forest), referred to as habitat (see Table S1). The proportion of use for each habitat was calculated by comparing the number of bear observations (GPS radiotelemetry fixes) in each habitat attribute with the total number of observations across all habitat types (equation 1 in Nielsen et al. 2013a). Each observation was taken from a 30 × 30-meter raster cell in arcgis (v9.x; Environmental Systems Research Institute (ESRI), Redlands, CA, USA) in accordance with previous grizzly bears studies (Northrup et al. 2012; Nielsen et al. 2013a). For the genetic analysis, DNA was isolated and fifteen microsatellites were amplified following Proctor et al. (2004). Hardy–Weinberg equilibrium was evaluated in genepop 4.0 (Rousset 2008) and homozygosity by loci (introduced by Aparicio et al. 2006) were calculated.

### Statistical analyses

All analyses were performed irrespective of management unit unless otherwise stated. Three approaches were used to identify genetic and habitat use clusters, with the proportional habitat use values transformed (arcsine-square-root) to account for overdispersion (Sokal and Rohlf 1995). First, we used K-Means clustering that assigns individuals to groups by maximizing the among-group sum of squared errors. Habitat distances among individuals were calculated by squaring the absolute difference in proportion of use. A simulated annealing algorithm was run for 5 000 000 steps and repeated 100 times from *k *=* *1 to *k *=* *20. Both the Bayesian information criteria (BIC) and pseudo-F were used to select the number of *k* (referred to as ecotype). Second, for the genotype data, we used structure 2.3.4 (Pritchard et al. 2000) to assess genetic structure independent of sampling area. We assumed an admixed model with correlated allele frequencies (Falush et al. 2003); one million iterations were run, with the first 20% omitted, and repeated three times. Individuals were assigned to a cluster based on their highest *Q* score. We selected these two approaches as they can produce discrete cluster assignments that could be directly contrasted. A Pearson correlation between individual ecotype/genotype *k* assignments was calculated in R2.15.0 (R Core Development Team 2012). The third strategy was a principal components analysis (PCA), based on a covariance matrix, which we used to identify the multivariate patterns of habitat use and genetic structure separately. The PCAs were implemented using the software genodive v2.0b22 (Meirmans and Van Tienderen 2004).

The relationship between gene flow and ecological distance, known as isolation-by-ecology (IBE; Shafer and Wolf 2013), was examined using Mantel tests (Mantel 1967). While this approach is typically applied between populations (Nosil 2012), individual-based approaches can be similarly used (Nielsen et al. 2013a), with a significant correlation taken as evidence for local adaptation only after the effect of geography has been partialed out of the model (Shafer and Wolf 2013). Here, we assessed the correlation between genetic relatedness and ecological divergence (i.e., differences in proportions of habitats used). Two genetic metrics (*G*) were used: the Queller and Goodnight (1989) pairwise relatedness and a coancestry matrix. The latter matrix was constructed using the software mol_coan v.3 (Fernández and Toro 2006) that employs a simulated annealing approach to create virtual common ancestors of the genotyped individuals and produces *pseudo* pedigree coefficients. Model parameters consisted of 300 steps with 5000 solutions tested per step, an initial temperature of 0.01 and increase of 0.75. We simulated two previous generations assuming 350 males and 350 females. The Euclidean distances (*D*) between individual capture locations was included to account for the confounding effects of eco-spatial autocorrelation and isolation-by-distance (Shafer and Wolf 2013). To account for the correlations due to sex and learning (Nielsen et al. 2013a), we constructed a matrix with female/female comparisons coded as 0, female/male as 0.5, and male/male as 1. The ecological matrix (*E*) was based on the absolute difference between individual PC scores on the first three axes of habitat use (denoted *E*^PC1-3^). The relationships among matrices and 95% confidence intervals were estimated from 10 000 permutations (‘ecodist’ R library).

To examine the variation in mass, SLL, and BCI, we fit hierarchical linear models in a Bayesian framework. We included four parameters we expected to influence these metrics: sex (bears are sexually dimorphic), age (older animals are expected to be larger than younger animals), capture location (body size is expected to increase with latitude), and capture season (bear weight can change substantially among seasons). In addition, we included covariates relevant to the current analysis, including individual homozygosity, *K* assignment (for both ecotype and genotype), and individual PC1 and 2 axis scores. Mass and SLL were log-transformed to ensure proper support. We fit a set of four models to each dependent variable starting with a base model of the five covariates predicted to influence fitness measures, and adding in genetic or habitat use variables (see Table S2). Models were compared using the deviance information criteria (DIC; Spiegelhalter et al. 2002), and convergence was assessed via the Gelman–Rubin diagnostic (values <1.1 indicating convergence to the posterior distribution; Gelman and Rubin 1992). All models were fit with intercepts varying by management unit. We fit two additional models for each metric with slopes for individual homozygosity varying by management unit to examine whether certain populations exhibited a different effect of homozygosity, and a homozygosity by sex interaction, which tested if there was differences in the effect of homozygosity according to sex. We calculated 95% credible intervals from the resulting posterior distributions for each coefficient. Specific model parameters and code are available in the supplementary material, and all analyses were run in R using the ‘rjags,’ ‘R2jags,’ and ‘coda’ libraries.

Finally, we obtained the translocation history for grizzly bears moved by management agencies in the province between 1974 and 2012 and determined the rate and direction of translocation among management units. We determined the amount of each habitat available within management unit by random sampling (by 30 × 30-meter raster cell) the multi-annual home ranges of each individual bear. A total of 150 000 random points were selected across our study system. Each random point was scored according to habitat class and averaged across the management unit. The absolute difference between each management unit was calculated, and a neighbor-joining tree was constructed using the population-habitat availability distance matrix (‘ape’ library in R).

## Results

A total of 88 grizzly bears had GPS radio collar and genetic data (Fig. 1). Over the course of 9 years (1999–2008), we collected 146 602 individual GPS locations that were used to calculate the proportion of use of seven habitat variables based on land cover classes (Table S3). Upland forest was the dominant habitat used by grizzly bears (~53% of locations), and wetland herbaceous was used least (<1%). Use of the remaining habitat types was evenly distributed. We genotyped individuals at 15 microsatellites and detected no deviations from Hardy–Weinberg equilibrium. There was an average of 8.5 alleles per locus with an observed heterozygosity of 0.67, and genotyping was >99% complete. Basic population genetic diversity statistics are provided in Table S4. Individual morphometric data are shown in Table S5: fewer (*n *=* *64) individuals had complete morphometric data and were used in the mixed-model analysis. Correlations between the three phenotypic variables ranged from 0.40 to 0.87.

**Figure 2:**
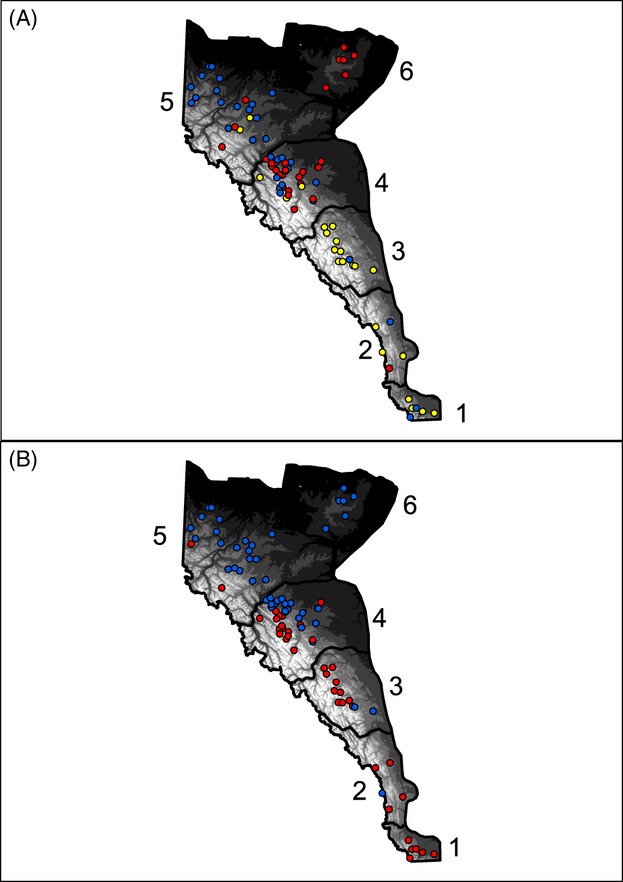
Map showing individual cluster assignments of grizzly bears (*Ursus arctos*) in Alberta, Canada. (A) structure-based assignment based on genetic data; (B) K-means assignment based on habitat use data. Six management units are identified: (1) Castle, (2) Livingstone, (3) Clearwater, (4) Yellowhead, (5) Grande Cache, and (6) Swan Hills.

For the K-means clustering, the Pseudo-F method suggested a *k* of 2 for habitat use (Fig. 2A), but the BIC could not resolve any cluster (*k *=* *20). structure supported a *k* of 3 (based on the Evanno et al. 2005 criteria) for the genetic data (Fig. 2B). Correlation among clusters (i.e., ecotype/genotype) for the genetic and habitat data assignments was moderate (Pearson *r *=* *0.34, *P *<* *0.01). The three main genetic axes of principal components (PCs) accounted for 7% 6%, and 5% of the variance, respectively (see Figure S1). The main habitat use axes accounted for 49%, 22%, and 13% of the individual variance (Figure S2; Table S6). The IBE correlations with interpretations are presented in Table 1. Notably, the strongest detectable relationship was between ecological distance (*E*^PC2^) and genetic coancestry (Table 1; Figure S2), such that individuals with similar habitat use patterns were genetically more related: this pattern showed no evidence of being confounded by sex-based differences or spatial autocorrelation.

**Table 1:** Mantel tests showing the correlation between genetic relatedness (*G*), ecological divergence (*E*), sex (*S*) and geographic distance (*D*) of 88 individual grizzly bears. Variables following | are controlled for in the model. Sex coding was 0 for female/female, 0.5 for male/female, 1 for male/male comparisons. Ecological divergence was the absolute difference between PC axes 1, 2, and 3 of individual habitat use scores. Two different genetic metrics are used: the QG relatedness coefficient and a coancestry matrix. Correlation values are bolded if one-side *P* value is 0.05 or less. Strict interpretations are provided.

	Null models		
	*r* Value	*r* Value	Interpretation
*S ˜* E^PC1^	−**0.11**	–	Sex-based differences in habitat use
*S ˜* E^PC2^	0.09	–	No sex-based differences in habitat use
*S ˜* E^PC3^	**0.15**	–	Sex-based differences in habitat use
E^PC1^ *˜ D*	**0.13**	–	Habitat use is spatially autocorrelated
E^PC2^ *˜ D*	0.07	–	Habitat use is not spatially autocorrelated
E^PC3^ *˜ D*	0.07	–	Habitat use is not spatially autocorrelated
	QG	Coancestry	
*G* ~ *D*	−**0.10**	−0.03	Isolation-by-distance
*G ˜ S*	−0.04	−0.05	No sex-biased dispersal
	Ecological divergence models		
*G* ~ E^PC1^	−**0.05**^PM^	−0.07	Signature of ecological selection (local adaptation)
*G* ~ E^PC2^	−0.03	−**0.13**^PM^	Signature of ecological selection (local adaptation)
*G* ~ E^PC3^	−0.05	−0.01	No signature of ecological selection
*G* ~ *D* | *E*+ S	**0.07**	−0.01	Remaining variance explained by geographic distance
*G* ~ *E*| *D * + *S*	−0.04	−**0.12**	Remaining variance explained by ecological divergence

Uppercase PM denotes ecological divergence values used in the partial Mantel test for the respective genetic distance measure.

**Figure 3:**
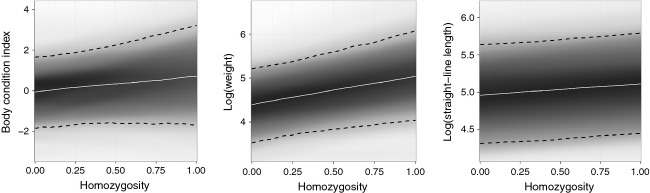
Visually weighted posterior predicted influence of homozygosity on body condition index, weight and straight-line length, from best models for each dependent variable as determined using the deviance information criteria. White lines represent the median response, and shading represents the uncertainty in the response, with darker areas indicating higher certainty. Ninety-five percent of the posterior prediction is denoted by the hashed line.

In the mixed-effects models, correlations among predictor variables were low (all |*r*| < 0.70) with the exception of capture location. Capture latitude and longitude were highly correlated due to grizzly bear distribution (Fig. 1), and thus, only latitude (Northing in Table 2) was examined. All models converged (i.e., Gelman–Rubin diagnostics were below 1.1), and we present the top three models with the lowest DIC (Table 2); all models are shown in Table S7. Bear sex and age were both significantly related to variation in all metrics (older bears and males generally were heavier, longer, and in better body condition). Capture season and longitude had no effect. All three fitness metrics were significantly influenced by ecotype, while body condition and mass were significantly influenced by habitat use PC1. Homozygosity had a significant positive effect on mass and length and was retained in the top model for all response variables (Table 2, Fig. 3). Allowing homozygosity to fluctuate over management units did not improve DIC scores, and all population-specific slopes were similar, suggesting a province-wide effect. The homozygosity correlation was not due to a single locus effect (*F = *1.13, df = 14, *P *=* *0.36; see Appendix S1).

**Table 2:** Results of top three model examining variation in mass, total length, and body condition for 64 individual grizzly bears. Bold indicates 95% credible intervals that did not overlap 0. For each model, *x* denotes and interaction term between sex and homozygosity (hom), where *y* allowed homozygosity to vary by management unit. PC1/2 is in reference to habitat use and genetic scores from the principal components analysis. Clusters (*k*) were categorical variables based on each individual's highest assignment: the genetic *k* was 3, so the two values reported are relative to the third cluster. Model number is in reference to Table S2.

									Genetics		Habitat			
Model no.	DIC	Sex	Age	Northing	Spring capture	Summer capture	% Hom	% Hom × Sex	*k*1/2	PC1/2	*k*	PC1/2	Overall intercept (*μ*_α_)	Overall homozygosity (*μ*_γ_)
Body condition														
1	163.5	**0.97**	**0.07**	0.16	0.49	0.68	–	–	–	–	–	–	−0.68	–
3	161.5	**0.68**	**0.07**	−0.12	0.41	0.6	0.78	–	−0.22/−0.42	–	**0.71**	–	−**1.65**	–
4	149.7	**0.70**	**0.07**	−0.12	0.41	0.46	0.81	–	–	−0.21/**0.36**		**1.48**/0.27	−0.76	–
Mass														
2	29.33	**0.55**	**0.05**	0.1	0.05	0.07	**0.63**	–	–	–	–	–	**3.92**	–
3	24.1	**0.45**	**0.05**	−0.6	0.01	0.005	**0.65**	–	−0.06/−0.07	–	**0.29**	–	**3.56**	–
4	24.6	**0.46**	**0.05**	−0.03	0.02	−0.04	0.56	–	–	−0.04/0.01	–	**0.45**/0.3		–
Length														
2	−148.1	**0.11**	**0.01**	−0.01	−0.01	−0.02	**0.15**	–	–	–	–	–	**4.89**	–
2^x^	−147.6	**0.17**	**0.10**	−0.002	−0.2	−0.03	**0.22**	−0.21	–	–	–	–	**4.87**	–
3	−146.2	**0.09**	**0.01**	−0.04	−0.02	−0.04	**0.15**	–	–0.01/0.0003	–	**0.05**	–	**4.82**	–

**Figure 4:**
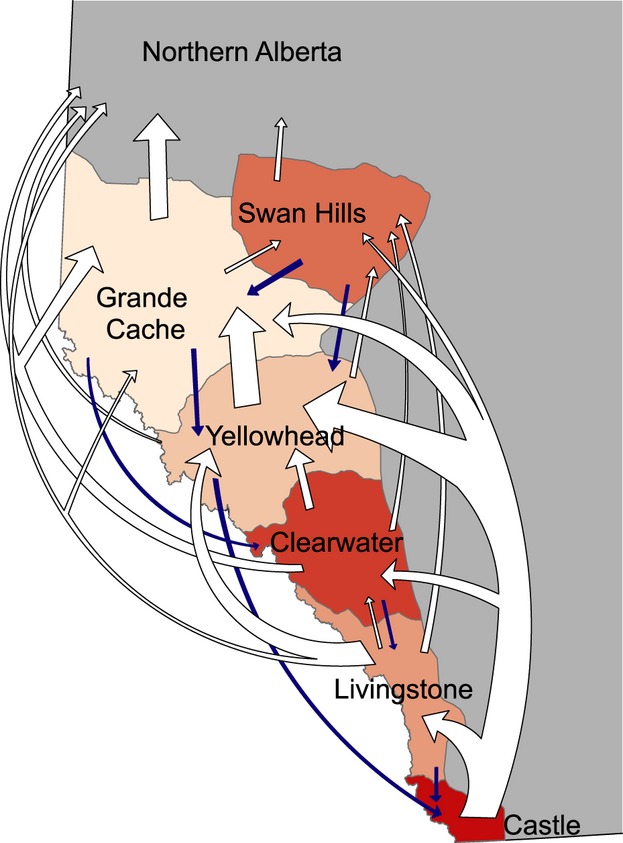
Map showing the translocations of grizzly bears (*Ursus arctos*) that have occurred in the province of Alberta between 1974 and 2012. The arrows are proportional to the number of animals moved.

**Figure 5:**
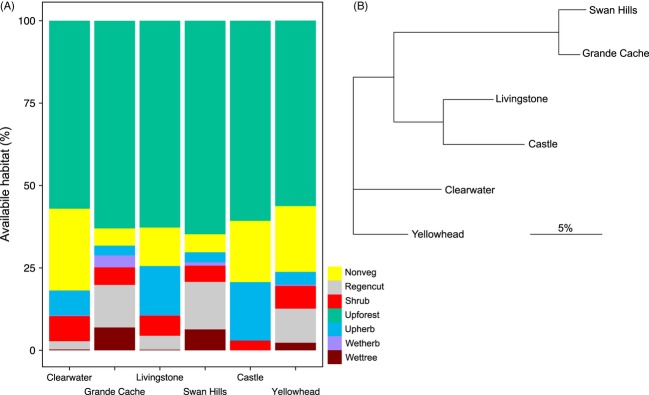
The proportion of the seven habitat classes available in each management unit (A). The absolute difference in habitat availability between management units displayed as a neighbor-joining tree (B).

Based on the translocation records available from 1974 to 2012, a total of 362 translocation events occurred within the province. Of these, 229 (63%) were translocations to different management units, of which 204 (~90%) were to a more northern management unit. The overall trends are visualized in Fig. 4. Habitat availability, based on land cover classes, differed significantly among management units (*χ*^2^ = 105.21, *P *<* *0.01; Fig. 5A). Among management units, the largest difference in habitat availability was observed between Castle and both Grande Cache and Swan Hills (~26%; Fig. 5B).

## Discussion

As expected, factors such as sex and age influenced fitness-associated traits in Alberta's grizzly bears, as did habitat use and genetic diversity. This is the first attempt to explicitly link genotype, ecotype, and phenotype in a wild carnivore. We also observed correlated genetic and habitat (ecotypes) clusters, and individual-based evidence for isolation-by-ecology (IBE), where ecological divergence is correlated with reduced gene flow. These patterns are consistent with ecologically mediated divergence or local adaptation. Surprisingly, we documented a positive effect of homozygosity on fitness traits (Fig. 3), most notably body mass and length. Finally, we reviewed 30 years of translocation data and showed an extremely high rate of human mediated movement, particularly to areas that differ significantly in available habitat (Figs 4 and 5).

### Linking genotype, ecotype, and phenotype

The link that exists between ecological and genetic divergence can have important implications for population demographics and adaptive divergence (Nosil 2007, 2012). In Alberta's grizzly bears, distinct genetic clusters and ecotypes have been previously suggested (Proctor and Paetkau 2004; Proctor et al. 2005; Munro et al. 2006; Nielsen et al. 2010; Coogan et al. 2012), and our results are in general accordance with these observations. Both the habitat clusters and principal component scores (Fig. 2B, S2a) show a distinction between the alpine and montane individuals, while the three genetic clusters are roughly broken down in a latitudinal fashion (Fig. 2A). The correlation between genotype and ecotype and the IBE pattern, taken as evidence for local adaptation (Nosil 2012; Shafer and Wolf 2013), has not previously been documented in this species. Moreover, dispersal of juveniles is unlikely to have a large effect on these metrics as recorded distances are small and likely within the delineated spatial clusters (average 42 km for males, 14 km for females; Proctor et al. 2004) and would only serve to lower ecotype:genotype correlations.

Habitat use also influenced grizzly bear phenotype, with both the ecotype and principal components influencing body mass and condition (Table 2). While it is not overly surprising that habitat use influenced body mass (see potential reasons in Allen et al. 2006), the collective analyses suggests that this pattern is partially adaptive, a result that is of evolutionary and ecological significance. Explicitly modeling these three components (genotype, ecotype, and phenotype) allowed for effect sizes and interactions to be estimated and are biologically relevant. Further, the adaptive divergence within Alberta's grizzly bear population is particularly important for management and conservation, primarily in regards to translocation decisions. Given the connection between body size and reproduction in this species (Stringham 1990), disrupting locally, co-adapted gene complexes could negatively influence fitness.

### Interpreting the negative HFC correlation

A major question remains: is there outbreeding depression in Alberta's grizzly bears, and if so, what is causing it? The impetus behind this study was to explore how differences in habitat use influenced fitness-associated traits. Given the literature on HFCs it was reasonable to include homozygosity as a covariate: however, we did not anticipate a negative HFC, let alone the magnitude of the effect, leading us to question the potential for outbreeding depression. Doubt may arise as to whether the findings we present are really evidence for such depression given sparse empirical evidence (Frankham et al. 2011). Here, the primary line of questioning should first revolve around the genetic data. Microsatellite datasets are often criticized for not reflecting genome-wide patterns of nuclear diversity (*r*^2^ of 0.70: Väli et al. 2008) or variation in inbreeding (Balloux et al. 2004). Despite caveats to such concerns and HFCs (Szulkin et al. 2010), we offer three lines of evidence that support the validity of this pattern: (i) the genotype:ecotype correlation and IBE pattern can be viewed as evidence for local adaptation, which is a requirement for negative HFCs; (ii) the homozygosity by loci measure is more reflective of genome-wide patterns of diversity and inbreeding than standard heterozygosity measures (Aparicio et al. 2006); (iii) the sex by homozygosity interaction had a steeper slope (stronger effect) in females, a pattern consistent with previous work on HFCs (Olano-Marin et al. 2011). Collectively, we view this as compelling evidence for a genuine negative HFC over a simple spurious pattern.

The second relevant question that must be answered is what processes could be causing this negative HFC? For this, we focused on the role of management-related translocation of bears, for which we were interested in quantifying the direction of translocations and difference in habitat availability among management units. The sheer number of translocations, particularly those being moved to areas with different habitat availability, is staggering, and a negative consequence on fitness would not be surprising (Robbins et al. 2004). The most common translocation route was from south (Castle) to north (Yellowhead), two areas that differ substantially in their available habitat (Figs 4 and 5). Under models of non-random gene flow (Edelaar and Bolnick 2012), we would predict this human-mediated movement to cause a reduction in fitness. Moreover, the level of translocation-induced gene flow far exceeds that which would be expected in an undisturbed system. Recent work showing learned habitat selection behaviors in grizzly bears cautioned against translocations because individuals may be unfamiliar with their new environment (Nielsen et al. 2013a), and studies have shown increased mortality rates with translocation (Linnell et al. 1997). It is conceivable that the HFC on mass, where outbred offspring have reduced body mass, might explain the decreased reproductive success of translocated grizzly bears (Miller and Ballard 1982; Brannon 1987). But for translocations to be truly implicated as the cause of a negative HFC, two things must occur: (i) translocated individuals reproduce with local individuals, and (ii) offspring from translocated and local parent crosses do relatively poorly in their new environment. To rebuke this hypothesis, a study tracking the lineage of translocated bears and testing for the inheritance of (maladaptive) habitat use patterns is warranted. Further, accounting for differences in diet in future models should be considered given the known influence of salmon on body size (Hilderbrand et al. 1999; McLellan 2011). Until such time, the patterns that we present here, coupled with the unprecedented human-mediated movement of bears in the province, highlight the need to consider the potential for genetic consequences of this intensive management.

An alternative explanation to the negative HFC involves trade-offs in life-history where a larger body size may not be optimal. For example, in lemon sharks, a negative relationship between heterozygosity and survival was observed (DiBattista et al. 2008); but fast growth and large body size were associated with reduced juvenile survival (DiBattista et al. 2007). Thus, traits traditionally viewed as beneficial can actually be detrimental, which in turn would invert the interpretation to a positive HFC. Being a large bear does appear disadvantageous when available protein is low (Welch et al. 1997; Rode et al. 2001). In Alberta, the phenological patterns of bear food differ between the alpine and montane groups (Nielsen et al. 2003, 2010; Coogan et al. 2012), thus the optimal body size may vary by local environment (McLellan 2011). Allowing homozygosity to vary by population did not improve the model fit, suggesting the negative HFC was a global pattern. Thus, for this scenario to be true, being large must be a province-wide limiting factor, which seems unlikely given the variation in protein availability (Mowat and Heard 2006) and diet (Robichaud 2009) in the province, and the general positive association between body size and reproductive success in grizzly bears (Stringham 1990). While bear life-history may account for some of this pattern, the influence of translocations and the traditional interpretation as a negative HFC appear equally valid at this point.

### Conservation and evolutionary implications

Given the threatened conservation status of Alberta's grizzly bears (Festa-Bianchet and Kansas 2010), the human-induced movement of genetic material (>200 bears, of a population of 700, moved to different management units over the last 30 years) is unprecedented, particularly considering they have been for societally driven management purposes, rather than for species conservation. Bear conflicts peak during years in which food is scarce (Mattson et al. 1992; Blanchard and Knight 1995) and individuals conditioned to anthropogenic food may have difficulties meeting dietary requirements in their new translocated environment (Robbins et al. 2004). Robbins et al. (2004) noted that managers often do not consider the quantitative aspects of bear diets and fail to match food resources between natal (or current home range) and translocated environments. This is a particularly relevant concern given the differences in habitat between management units in Alberta (Fig. 5). While the pattern we report for outbreeding depression is not conclusive, the evidence for local adaptation coupled with non-natural migration (i.e., translocations), leads us to hypothesize translocations are causing the negative HFC. More than 10% of the population has been sampled in this study, but the logistical and financial issues of sampling more bears in the manner required for this analysis likely precludes more detailed information than is presented here.

Considering these factors together, we find it plausible that current management practices are having negative long-term fitness consequences on the population. While short-term effects of captures are known (Cattet et al. 2008; Nielsen et al. 2013b), these data suggest additional evolutionary implications to translocations, and the potential for such consequences should not be taken lightly. While theoretical work suggests the probability of outbreeding depression being low, what constitutes *meaningful environmental differences* is vague at best (Frankham et al. 2011). Perhaps the levels of local adaptation and differences in habitat purported here are enough. The evidence for learned habitat selection in this species (Nielsen et al. 2013a) and nutritional considerations of problem bears (Robbins et al. 2004) would suggest that translocated females are at a disadvantage in nourishing and teaching their cubs where to forage. The unexpected negative HFC should be explored further with particular emphasis on the effect of current management practices and grizzly bear life history. In conclusion, While we have shown that multiple factors influence fitness proxies of free-ranging grizzly bears including an unexpected negative HFC, whether the latter is caused by human-induced movement, while provocative, remains unsubstantiated.

## Data archiving statement

Genotypes have been deposited in the Dryad Digital Repository: http://www.dx.doi.org/10.5061/dryad.c4j84

## Supporting Information

**Figure S1**. Map showing individual PC1-3 scores of grizzly bears (*Ursus* arctos) in Alberta, Canada: (a–c) based on genotypic data; (d–f) based on habitat-use data.

**Figure S2**. Map showing individual PC1-3 scores of grizzly bears (*Ursus* arctos) in Alberta, Canada: (a–c) based on habitat-use data.

**Figure S3**. Plots showing the relationship between (a) coancestry and geographic distance; (b) relatedness (QG) and geographic distance; (c) coancestry and ecological distance (PC2), and (d) relatedness and ecological distance (PC1).

**Appendix S1**. Linear mixed modeling.

**Table S1**. The seven land cover classes (referred to as habitat) for which the proportion of use was calculated for each individual grizzly bear (*Ursus arctos*). **Table S2**. Candidate models fit to the three condition metrics (mass, length and body condition). **Table S3**. Proportion of use for each habitat variable according to individual (*n* = 88). **Table S4**. Basic population genetic diversity statistics and the standard error (SE) for each management unit for grizzly bears (*Ursus arctos*) in Alberta, Canada (*n* = 88). **Table S5**. Individual morphometric data (*n* = 64) and homozygosity. **Table S6**. The first three principal component (PC) axes of habitat use in grizzly bears (*Ursus arctos*) in Alberta, Canada. **Table S7**. Model results examining variation in mass, total length, and body condition.
