# Supplementary figures and images for: Linking genotype, ecotype, and phenotype in an intensively managed large carnivore
Source: Evol Appl. 2013 Dec 4;7(2):301–12. doi: 10.1111/eva.12122 (PMC3927890; doi:10.1111/eva.12122)

a)

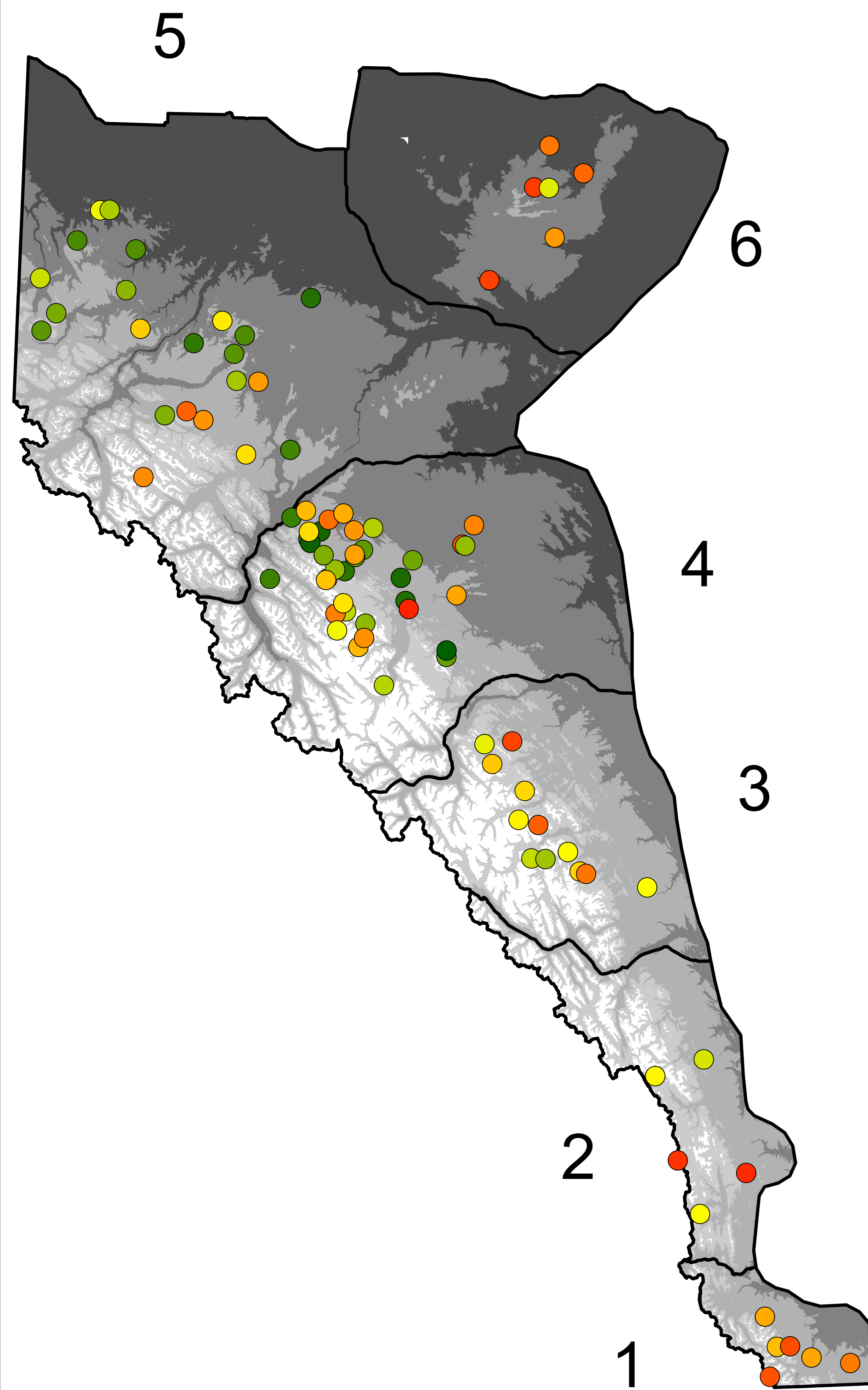

b)

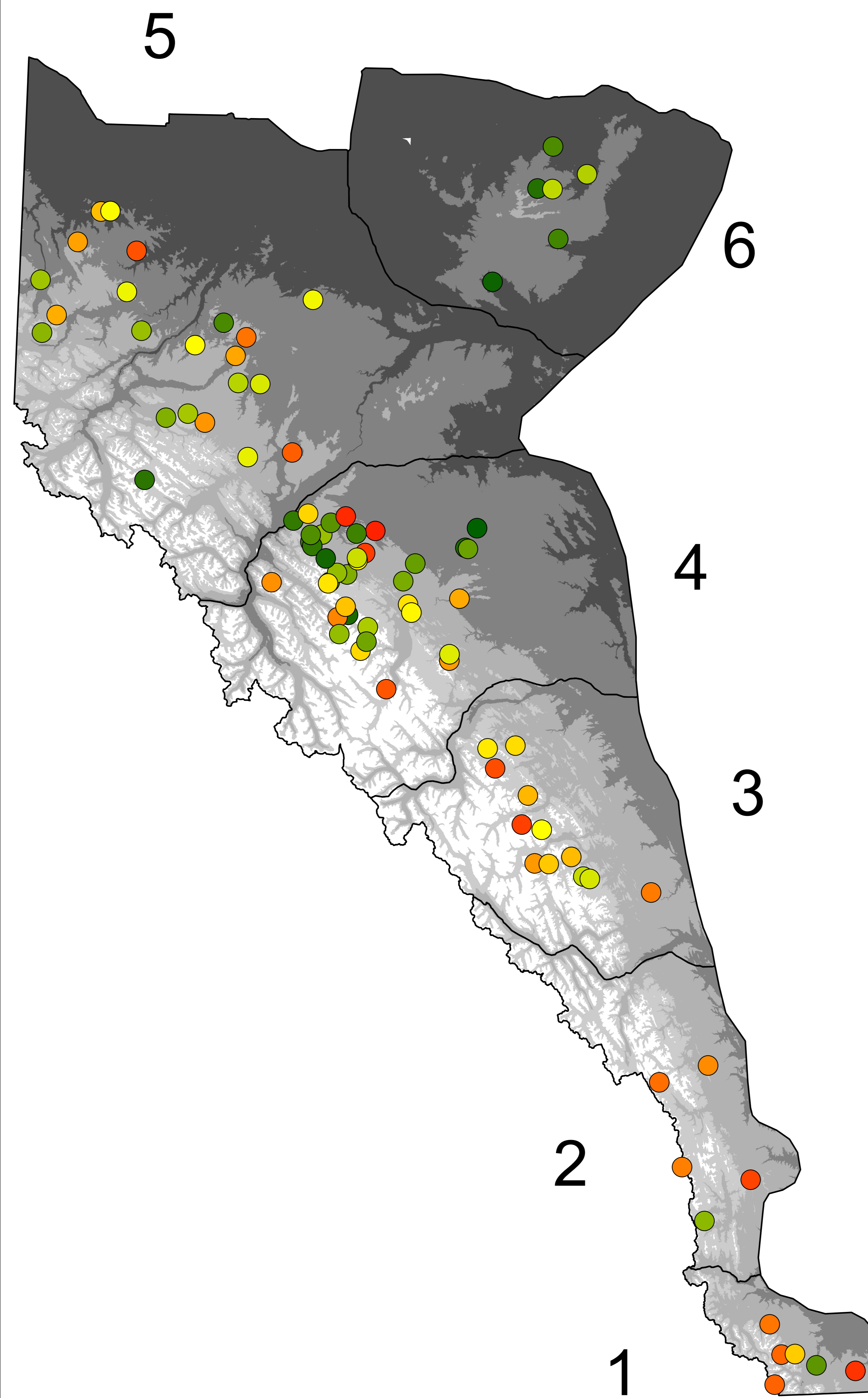

c)

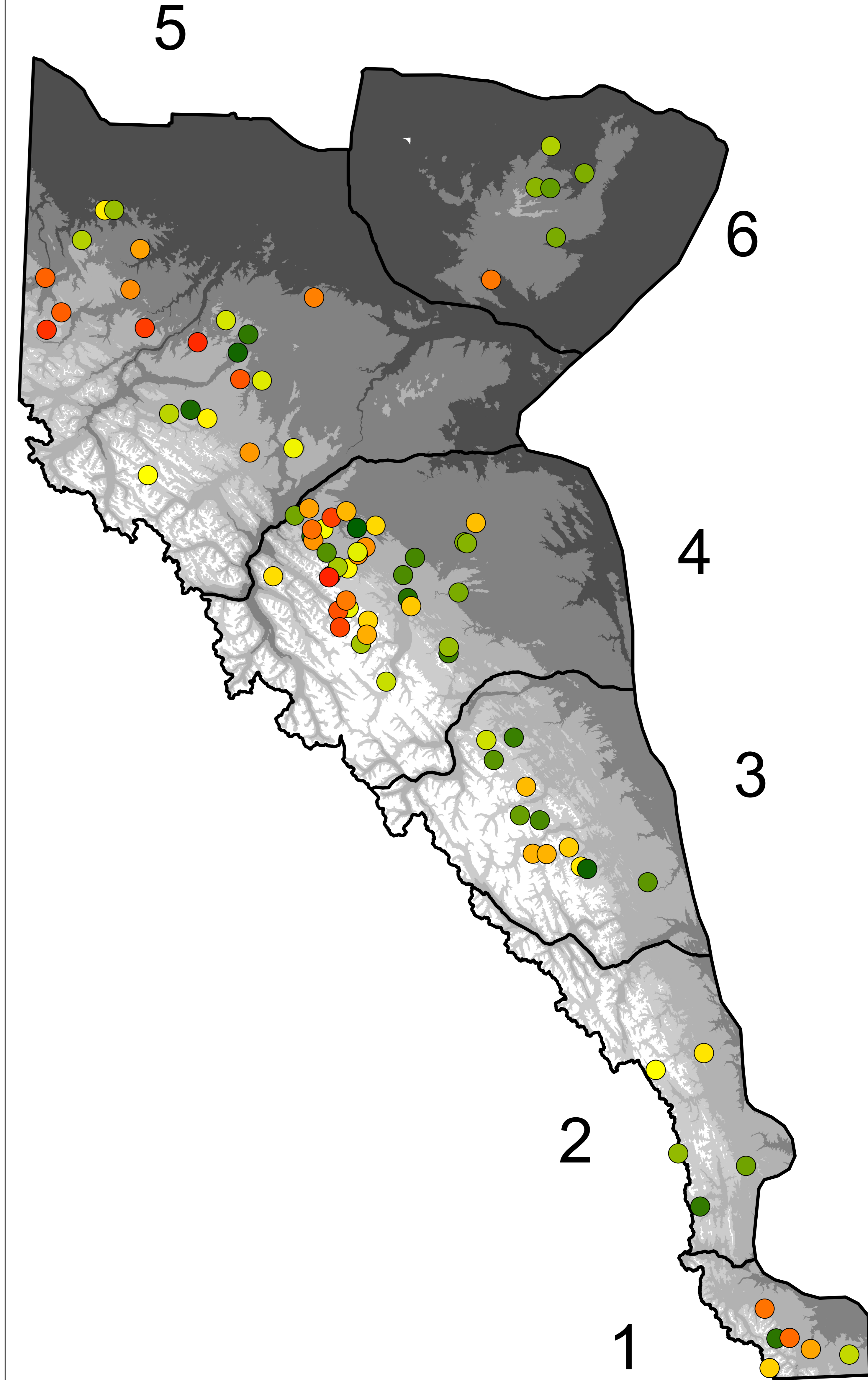

Supplement: Supplementary file 1 — Figure S1. Map showing individual PC1-3 scores of grizzly bears (Ursus arctos) in Alberta, Canada: (a–c) based on genotypic data; (d–f) based on habitat-use data. [file eva0007-0301-sd1.pdf]

a)

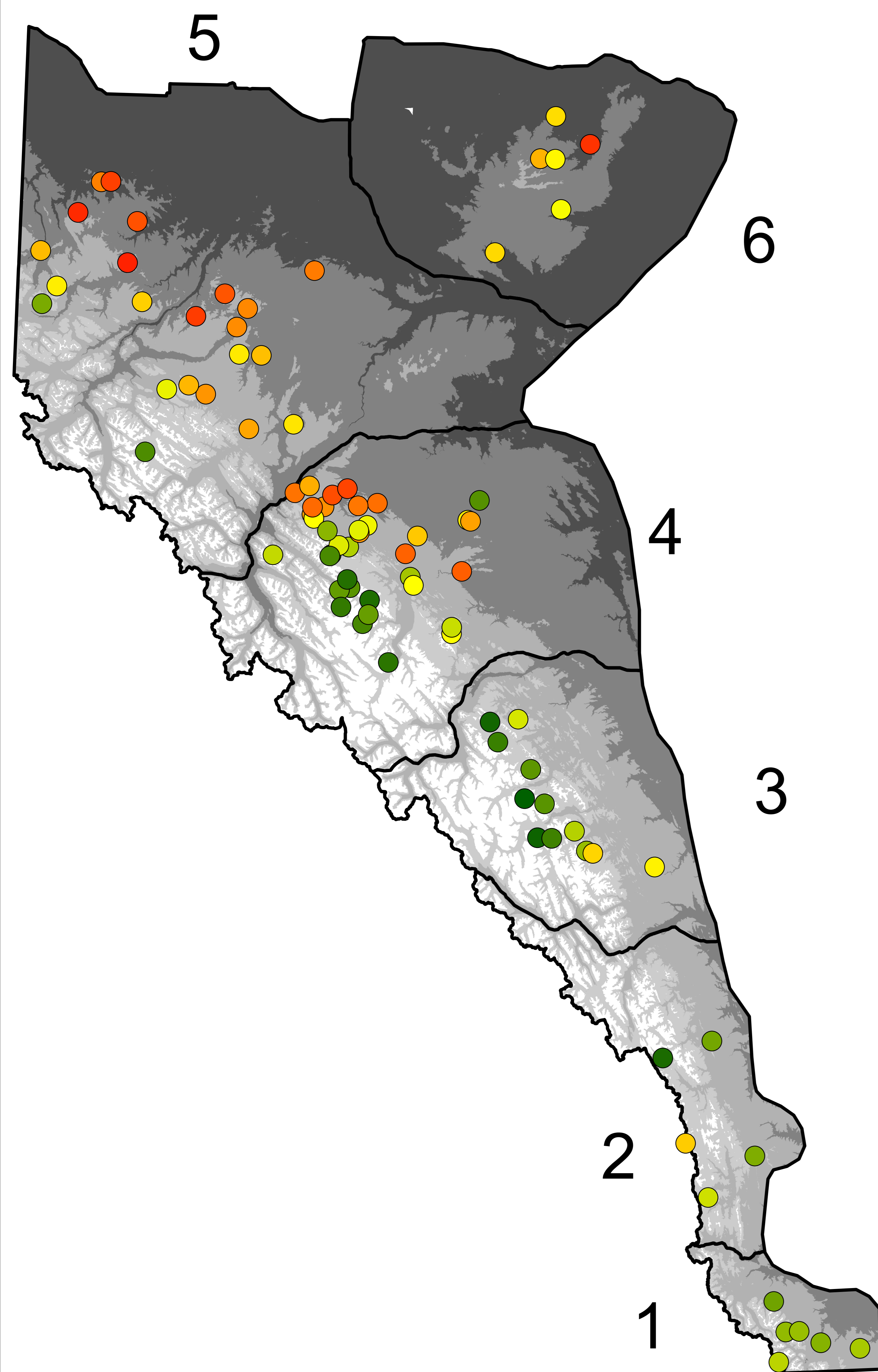

b)

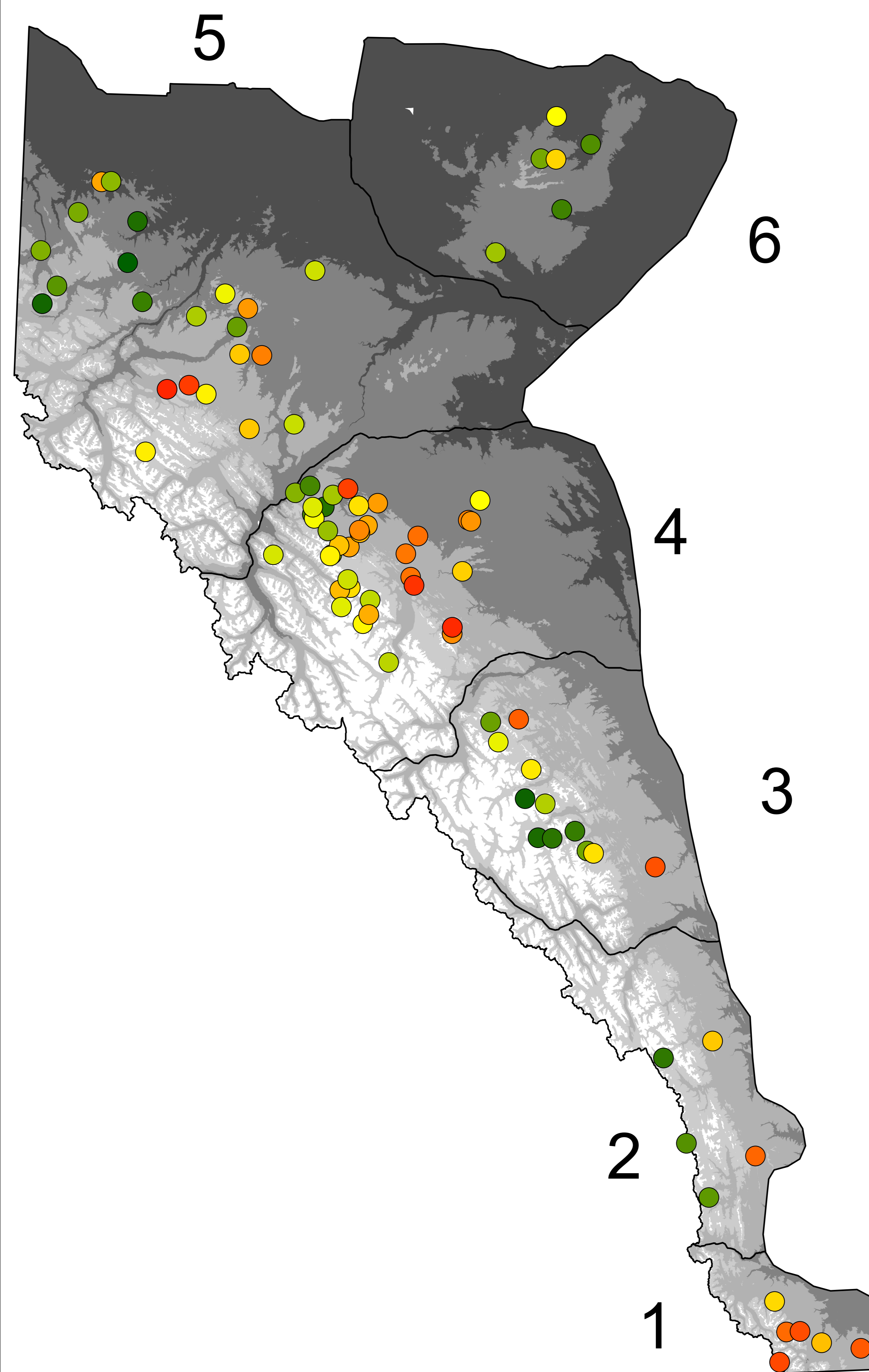

c)

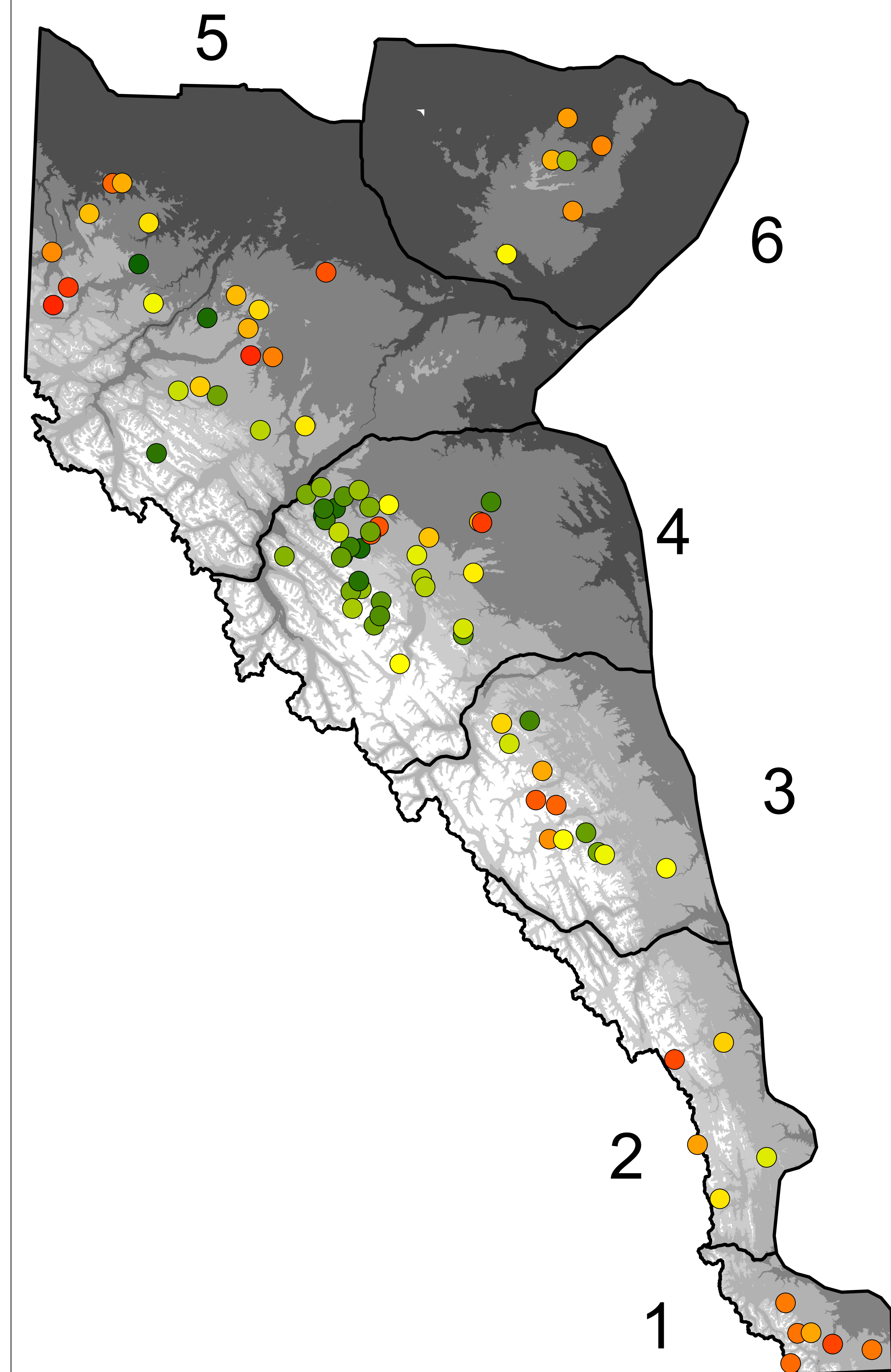

Supplement: Supplementary file 2 — Figure S2. Map showing individual PC1-3 scores of grizzly bears (Ursus arctos) in Alberta, Canada: (a–c) based on habitat-use data. [file eva0007-0301-sd2.pdf]

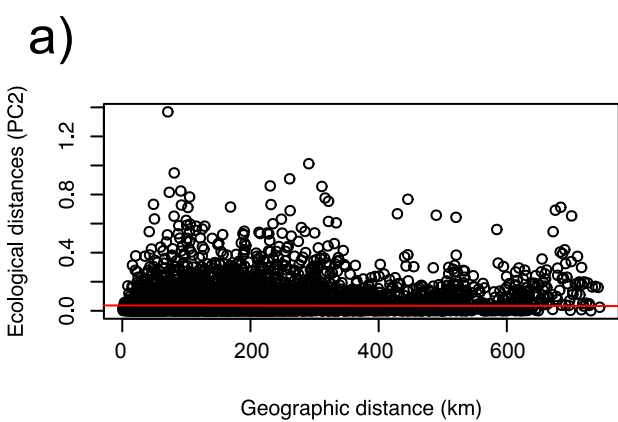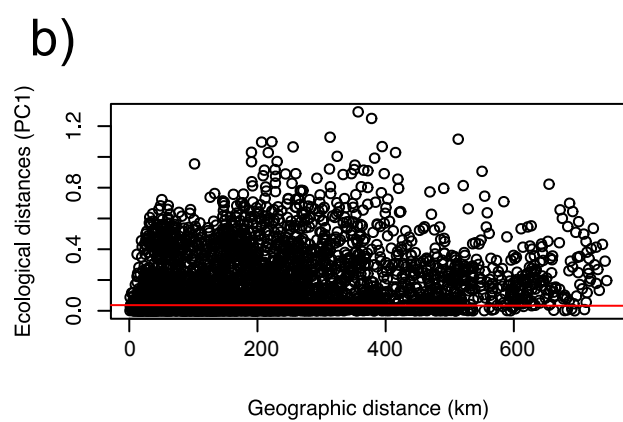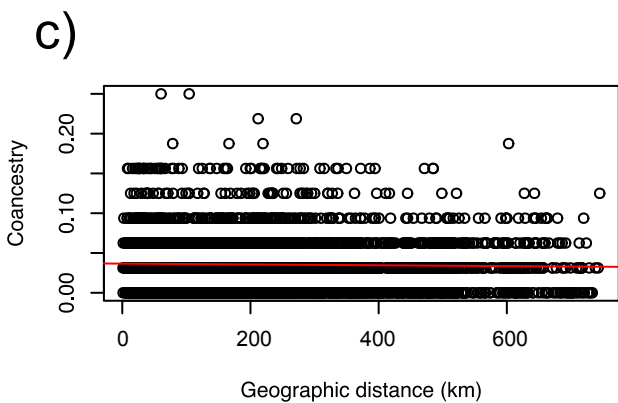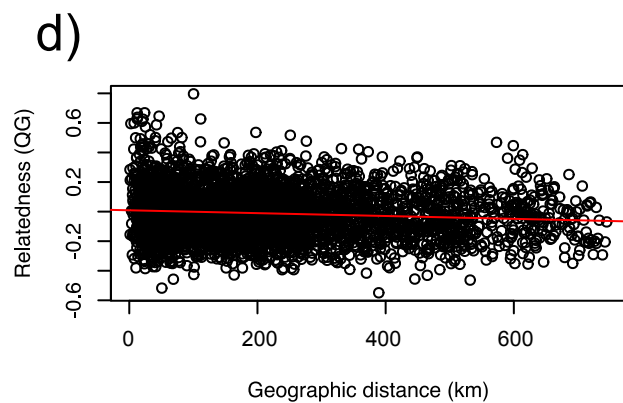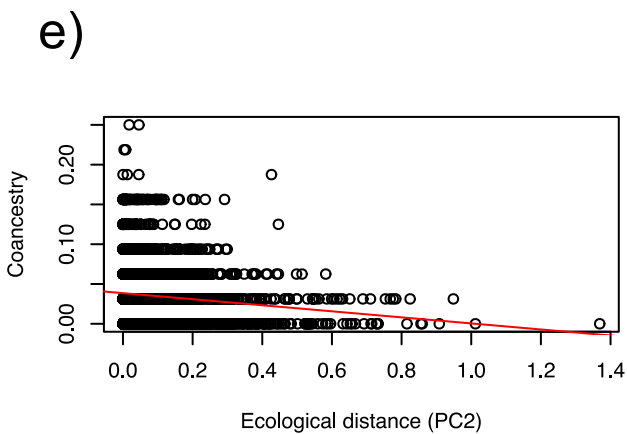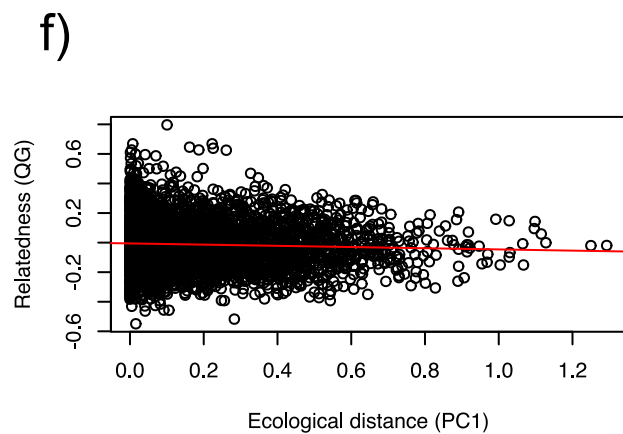

Supplement: Supplementary file 3 — Figure S3. Plots showing the relationship between (a) coancestry and geographic distance; (b) relatedness (QG) and geographic distance; (c) coancestry and ecological distance (PC2), and (d) relatedness and ecological distance (PC1). [file eva0007-0301-sd3.pdf]
