# Supplementary material for: Linking genotype, ecotype, and phenotype in an intensively managed large carnivore
Source: Evol Appl. 2013 Dec 4;7(2):301–12. doi: 10.1111/eva.12122 (PMC3927890; doi:10.1111/eva.12122)
Supplement: Supplementary file 4 — Appendix S1. Linear mixed modeling. [file eva0007-0301-sd4.doc]

**Linear mixed modeling.** We used vague normal priors (mean=0 and variance=1,000) for all coefficients, and vague gamma priors on
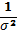
 for all variance parameters. The varying intercept and slope model takes the following form:


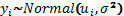


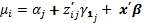


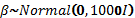


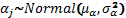


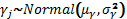


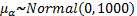


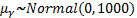


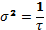


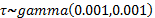


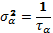


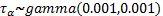


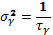


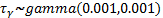


Where
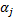
 is the intercept for the jth population,
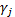
 is the coefficient for heterozygosity for the jth population, and
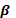
is a vector of coefficients for the remaining variables
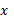
. We ran two chains of 1,000,000 iterations, discarding the first 500,000 as burn-in and assessed convergence to the posterior distribution by examining traceplots and using the Gelman-Rubin diagnostic (
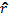
; Gelman and Rubin 1992). For model 4 (Supplemental Table 1), all parameters had not converged (
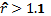
) at 1,000,000 iterations, so we ran this model for 2,000,000 iterations, discarding the first 1,000,000 as burn-in.

#Jags model to run linear regression with random slope and random intercept

model{

#priors

mu.b0~dnorm(0,0.00001)

tau.b0~dgamma(0.001,001)

mu.b1~dnorm(0,0.00001)

tau.b1~dgamma(0.001,001)

tau.y~dgamma(0.001,0.001)

beta[1:n.beta]~dmnorm(mu.beta[], omega.beta[,])

#population betas

for(j in 1:n.pop){

b0[j]~dnorm(mu.b0, tau.b0)

b1[j]~dnorm(mu.b1, tau.b1)

}

#model

for(i in 1:length(y)){

mu[i] <- b0[pop[i]] + b1[pop[i]]*x6[i]+beta[1]*x1[i] + beta[2]*x2[i] + beta[3]*x3[i] + beta[4]*x4[i] + beta[5]*x5[i] #+ beta[6]*x6[i]

y[i] ~ dnorm(mu[i],tau.y)

}

}

**Single locus effect of heterozygosity-fitness correlations.** To test for a single locus effect we followed the methods of Szulkin et al. (2010). Each locus was treated as a fixed effect and was coded 1 for heterozygote and 0 for homozygote. An *F*-ratio test (amova.lm argument in *R*) was used to compare the single locus model to the average homozygosity model (the null hypothesis is that all loci equally contribute to the HFC).
