# Supplementary material for: Linking genotype, ecotype, and phenotype in an intensively managed large carnivore
Source: Evol Appl. 2013 Dec 4;7(2):301–12. doi: 10.1111/eva.12122 (PMC3927890; doi:10.1111/eva.12122)
Supplement: Supplementary file 5 — Table S1. The seven land cover classes (referred to as habitat) for which the proportion of use was calculated for each individual grizzly bear (Ursus arctos). Table S2. Candidate models fit to the three condition metrics (mass, length and body condition). Table S3. Proportion of use for each habitat variable according to individual (n = 88). Table S4. Basic population genetic diversity statistics and the standard error (SE) for each management unit for grizzly bears (Ursus arctos) in Alberta, Canada (n = 88). Table S5. Individual morphometric data (n = 64) and homozygosity. Table S6. The first three principal component (PC) axes of habitat use in grizzly bears (Ursus arctos) in Alberta, Canada. Table S7. Model results examining variation in mass, total length, and body condition. [file eva0007-0301-sd5.docx]

**Table S1.** The seven land cover classes (referred to as habitat) for which the proportion of use was calculated for each individual grizzly bear (*Ursus arctos*).

|  |  |
| --- | --- |
| Habitat variable | Description |
| wettree | wetland treed |
| regencut | regenerating cutblocks |
| shrub | shrubs |
| wetherb | wetland herbaceous |
| upherb | upland herbaceous |
| nonveg | barren landcover |
| upforest | upland forest |

**Table S2**. Candidate models fit to the three condition metrics (mass, length and body condition).

| Model | Covariates with fixed slopes | Covariates with varying slopes by management unit |
| --- | --- | --- |
| 1 | Sex + age + latitude + capture season | Intercept |
| 2 | Sex + age + latitude + capture season + homozygosity | Intercept & homozygosity |
| 3 | Sex + age + latitude + capture season + homozygosity + *K* clusters | Intercept |
| 4 | Sex + age + latitude + capture season + homozygosity + PC scores | Intercept |

**Table S3.** Proportion of use for each habitat variable according to individual (*n* = 88). Table S5 describes all the habitat variables. Note, these values have not been transformed.

| Individual | nonveg | regencut | shrub | uplndherb | uplndtree | wtlndherb | wtlndtree |
| --- | --- | --- | --- | --- | --- | --- | --- |
| G001 | 0.117 | 0 | 0.354 | 0.093 | 0.435 | 0 | 0 |
| G002 | 0.146 | 0 | 0.257 | 0.224 | 0.374 | 0 | 0 |
| G003 | 0.095 | 0 | 0.301 | 0.116 | 0.488 | 0 | 0 |
| G004 | 0.093 | 0.003 | 0.411 | 0.162 | 0.332 | 0 | 0 |
| G006 | 0.075 | 0 | 0.306 | 0.105 | 0.514 | 0 | 0 |
| G007 | 0.151 | 0.029 | 0.084 | 0.031 | 0.621 | 0.005 | 0.078 |
| G008 | 0.025 | 0.025 | 0.319 | 0.052 | 0.579 | 0 | 0 |
| G010 | 0.110 | 0 | 0.324 | 0.166 | 0.400 | 0 | 0 |
| G011 | 0.006 | 0.136 | 0.072 | 0.014 | 0.706 | 0.011 | 0.056 |
| G012 | 0.011 | 0.145 | 0.067 | 0.023 | 0.677 | 0.006 | 0.072 |
| G013 | 0.074 | 0.001 | 0.408 | 0.089 | 0.427 | 0 | 0 |
| G014 | 0.041 | 0.087 | 0.053 | 0.025 | 0.618 | 0.031 | 0.145 |
| G017 | 0.024 | 0.232 | 0.072 | 0.034 | 0.620 | 0.005 | 0.012 |
| G020 | 0.022 | 0.372 | 0.126 | 0.079 | 0.393 | 0.005 | 0.004 |
| G023 | 0.058 | 0.062 | 0.238 | 0.048 | 0.593 | 0 | 0 |
| G024 | 0.015 | 0.349 | 0.058 | 0.045 | 0.518 | 0.002 | 0.013 |
| G027 | 0.041 | 0.046 | 0.089 | 0.072 | 0.711 | 0.008 | 0.034 |
| G028 | 0.07 | 0 | 0.414 | 0.183 | 0.332 | 0 | 0 |
| G033 | 0.032 | 0.077 | 0.080 | 0.026 | 0.712 | 0.016 | 0.058 |
| G034 | 0.056 | 0 | 0.355 | 0.084 | 0.505 | 0 | 0 |
| G035 | 0.095 | 0 | 0.35 | 0.115 | 0.44 | 0 | 0 |
| G036 | 0.064 | 0.305 | 0.083 | 0.030 | 0.494 | 0.002 | 0.021 |
| G037 | 0.032 | 0.350 | 0.150 | 0.049 | 0.415 | 0 | 0.004 |
| G038 | 0.05 | 0.151 | 0.185 | 0.064 | 0.55 | 0 | 0 |
| G040 | 0.172 | 0.040 | 0.227 | 0.127 | 0.434 | 0 | 0 |
| G043 | 0.018 | 0.003 | 0.251 | 0.091 | 0.622 | 0.003 | 0.012 |
| G044 | 0.038 | 0 | 0.476 | 0.163 | 0.322 | 0 | 0 |
| G045 | 0.026 | 0.038 | 0.026 | 0.038 | 0.628 | 0.026 | 0.218 |
| G048 | 0.18 | 0.067 | 0.206 | 0.057 | 0.49 | 0 | 0 |
| G050 | 0.038 | 0.306 | 0.086 | 0.115 | 0.431 | 0.005 | 0.019 |
| G054 | 0.016 | 0.306 | 0.097 | 0.032 | 0.54 | 0 | 0.008 |
| G055 | 0.02 | 0.027 | 0.246 | 0.041 | 0.643 | 0.005 | 0.018 |
| G058 | 0.01 | 0.016 | 0.138 | 0.018 | 0.802 | 0 | 0.016 |
| G059 | 0.16 | 0 | 0.032 | 0.053 | 0.755 | 0 | 0 |
| G060 | 0.01 | 0.165 | 0.019 | 0 | 0.796 | 0 | 0.01 |
| G062 | 0.045 | 0.066 | 0.177 | 0.024 | 0.68 | 0 | 0.008 |
| G064 | 0.296 | 0 | 0.016 | 0.107 | 0.58 | 0 | 0 |
| G065 | 0.03 | 0 | 0.150 | 0.022 | 0.792 | 0 | 0.006 |
| G066 | 0.009 | 0.198 | 0.126 | 0.036 | 0.568 | 0 | 0.063 |
| G068 | 0.03 | 0.163 | 0.104 | 0.022 | 0.630 | 0 | 0.052 |
| G071 | 0.128 | 0 | 0.229 | 0.321 | 0.321 | 0 | 0 |
| G072 | 0.023 | 0.019 | 0.240 | 0.032 | 0.684 | 0 | 0.001 |
| G073 | 0.218 | 0 | 0.170 | 0.115 | 0.497 | 0 | 0 |
| G074 | 0.077 | 0 | 0.266 | 0.238 | 0.402 | 0 | 0 |
| G076 | 0.203 | 0 | 0.054 | 0.074 | 0.668 | 0 | 0 |
| G077 | 0.05 | 0 | 0.062 | 0.147 | 0.742 | 0 | 0 |
| G078 | 0.011 | 0 | 0.149 | 0.201 | 0.639 | 0 | 0 |
| G080 | 0.083 | 0.227 | 0 | 0.212 | 0.477 | 0 | 0 |
| G081 | 0.208 | 0 | 0.092 | 0.153 | 0.547 | 0 | 0 |
| G086 | 0.184 | 0.105 | 0.092 | 0.17 | 0.449 | 0 | 0 |
| G087 | 0.029 | 0 | 0.023 | 0.224 | 0.724 | 0 | 0 |
| G088 | 0.025 | 0 | 0.111 | 0.321 | 0.543 | 0 | 0 |
| G089 | 0.139 | 0 | 0.072 | 0.509 | 0.280 | 0 | 0 |
| G091 | 0.294 | 0 | 0.228 | 0.262 | 0.215 | 0 | 0 |
| G092 | 0.359 | 0 | 0.163 | 0.313 | 0.165 | 0 | 0 |
| G093 | 0.201 | 0 | 0.157 | 0.225 | 0.399 | 0.007 | 0.010 |
| G095 | 0.098 | 0.065 | 0.228 | 0.19 | 0.418 | 0 | 0 |
| G096 | 0.064 | 0.140 | 0.098 | 0.06 | 0.613 | 0.004 | 0.021 |
| G097 | 0.172 | 0.014 | 0.257 | 0.27 | 0.287 | 0 | 0 |
| G098 | 0.024 | 0.033 | 0.102 | 0.057 | 0.765 | 0.005 | 0.014 |
| G099 | 0.129 | 0.113 | 0.212 | 0.18 | 0.365 | 0 | 0 |
| G106 | 0.032 | 0.194 | 0.050 | 0.019 | 0.644 | 0.023 | 0.037 |
| G200 | 0.128 | 0.219 | 0.056 | 0.053 | 0.491 | 0.008 | 0.045 |
| G201 | 0.064 | 0.110 | 0.084 | 0.090 | 0.573 | 0.012 | 0.067 |
| G203 | 0.049 | 0.153 | 0.149 | 0.082 | 0.48 | 0.031 | 0.055 |
| G204 | 0.031 | 0.095 | 0.147 | 0.093 | 0.62 | 0.003 | 0.010 |
| G205 | 0.047 | 0.314 | 0.014 | 0.050 | 0.430 | 0.020 | 0.126 |
| G206 | 0.021 | 0.202 | 0.059 | 0.043 | 0.548 | 0.021 | 0.106 |
| G207 | 0.049 | 0.138 | 0.069 | 0.039 | 0.475 | 0.033 | 0.197 |
| G208 | 0.043 | 0.121 | 0.179 | 0.080 | 0.471 | 0.022 | 0.085 |
| G209 | 0 | 0 | 0.062 | 0.016 | 0.922 | 0 | 0 |
| G210 | 0.026 | 0 | 0.450 | 0.114 | 0.406 | 0.004 | 0 |
| G211 | 0.120 | 0.127 | 0.139 | 0.137 | 0.389 | 0.021 | 0.067 |
| G216 | 0.053 | 0.075 | 0.031 | 0.047 | 0.727 | 0.007 | 0.060 |
| G217 | 0.010 | 0.133 | 0.056 | 0.042 | 0.692 | 0.010 | 0.056 |
| G218 | 0.070 | 0.240 | 0.058 | 0.076 | 0.472 | 0.023 | 0.061 |
| G219 | 0.015 | 0.421 | 0.035 | 0.015 | 0.514 | 0 | 0 |
| G225 | 0.041 | 0.344 | 0.055 | 0.077 | 0.380 | 0.041 | 0.061 |
| G226 | 0.021 | 0.706 | 0.045 | 0.018 | 0.185 | 0.005 | 0.020 |
| G227 | 0.036 | 0.209 | 0.144 | 0.129 | 0.396 | 0.043 | 0.043 |
| G228 | 0.28 | 0 | 0.087 | 0.213 | 0.182 | 0.118 | 0.12 |
| G229 | 0.074 | 0.173 | 0.061 | 0.104 | 0.483 | 0.028 | 0.077 |
| G230 | 0.152 | 0.064 | 0.063 | 0.106 | 0.397 | 0.095 | 0.123 |
| G231 | 0.005 | 0.044 | 0.053 | 0.021 | 0.812 | 0.003 | 0.062 |
| G232 | 0.054 | 0.007 | 0.047 | 0.079 | 0.538 | 0.050 | 0.226 |
| G235 | 0.009 | 0.071 | 0.074 | 0.018 | 0.589 | 0.074 | 0.166 |
| G236 | 0.005 | 0.234 | 0.082 | 0.027 | 0.416 | 0.063 | 0.174 |
| G237 | 0.004 | 0.310 | 0.034 | 0.027 | 0.431 | 0.022 | 0.173 |

**Table S4.** Basic population genetic diversity statistics and the standard error (SE) for each management unit for grizzly bears (*Ursus arctos*) in Alberta, Canada (*n* = 88). Columns are: N – number of individuals; Na – number of alleles; Ho – observed heterozygosity; UHe – unbiased estimated heterozygosity; F – fixation index.

| Management Unit |  | N | Na | Ho | UHe | F |
| --- | --- | --- | --- | --- | --- | --- |
| Yellowhead | Mean | 37.00 | 6.87 | 0.69 | 0.69 | -0.02 |
|  | (SE) | (0.11) | (0.40) | (0.02) | (0.03) | (0.02) |
| Castle | Mean | 6.00 | 4.73 | 0.61 | 0.67 | 0.01 |
|  | (SE) | (0.00) | (0.34) | (0.05) | (0.04) | (0.06) |
| Swan Hills | Mean | 6.00 | 4.40 | 0.61 | 0.63 | -0.07 |
|  | (SE) | (0.00) | (0.36) | (0.06) | (0.05) | (0.06) |
| Livingstone | Mean | 5.00 | 4.00 | 0.64 | 0.65 | -0.11 |
|  | (SE) | (0.13) | (0.38) | (0.07) | (0.05) | (0.06) |
| Grande Cache | Mean | 22.00 | 6.33 | 0.68 | 0.66 | -0.04 |
|  | (SE) | (0.00) | (0.40) | (0.05) | (0.04) | (0.04) |
| Clearwater | Mean | 12.00 | 5.40 | 0.70 | 0.68 | -0.06 |
|  | (SE) | (0.00) | (0.29) | (0.04) | (0.03) | (0.03) |

**Table S5.** Individual morphometric data (*n* = 64) and homozygosity. Weight is in kilograms, straight-line length (SLL) is in centimeters, and the body-condition index (BCI) is a metric combining both weight and SLL.

| Individual | Weight | SLL | BCI | Homozygosity |
| --- | --- | --- | --- | --- |
| G002 | 78 | 152 | -0.63515 | 0.19 |
| G003 | 83.9 | 154 | -0.48437 | 0.26 |
| G004 | 76.2 | 155 | -1.05763 | 0.29 |
| G006 | 210.4 | 174 | 2.1179 | 0.37 |
| G010 | 90.7 | 154 | -0.10305 | 0.18 |
| G011 | 126.1 | 161 | 0.81412 | 0.52 |
| G013 | 70.3 | 164 | -2.35019 | 0.38 |
| G014 | 287.6 | 191 | 2.20104 | 0.45 |
| G017 | 216.8 | 177 | 1.99764 | 0.42 |
| G020 | 98.9 | 166 | -0.86349 | 0.26 |
| G023 | 107 | 160 | 0.10564 | 0.15 |
| G024 | 155.1 | 181 | -0.0154 | 0.33 |
| G027 | 93.4 | 156 | -0.16236 | 0.25 |
| G028 | 87.1 | 153 | -0.19879 | 0.26 |
| G033 | 105.2 | 159 | 0.12109 | 0.32 |
| G035 | 35.4 | 126 | -1.52925 | 0.26 |
| G036 | 67.1 | 141 | -0.19107 | 0.32 |
| G037 | 61.68 | 131 | 0.54353 | 0.40 |
| G040 | 78 | 149 | -0.32186 | 0.62 |
| G043 | 73.5 | 143 | 0.03187 | 0.39 |
| G045 | 212.2 | 181 | 1.53926 | 0.17 |
| G048 | 58.96 | 130 | 0.44426 | 0.11 |
| G050 | 134.24 | 161 | 1.12133 | 0.42 |
| G054 | 131.52 | 163 | 0.82702 | 0.31 |
| G055 | 97.98 | 152 | 0.47952 | 0.18 |
| G058 | 94.3 | 153 | 0.18963 | 0.26 |
| G059 | 118.8 | 149 | 1.73117 | 0.31 |
| G060 | 210 | 176 | 1.929 | 0.35 |
| G062 | 158.7 | 176 | 0.5431 | 0.19 |
| G064 | 86.2 | 146 | 0.4837 | 0.00 |
| G066 | 178.7 | 178 | 0.95183 | 0.32 |
| G071 | 91.6 | 158 | -0.45847 | 0.30 |
| G073 | 82.5 | 153 | -0.46413 | 0.60 |
| G074 | 85.3 | 145 | 0.53973 | 0.39 |
| G076 | 189.6 | 165 | 2.43521 | 0.41 |
| G077 | 88.9 | 148 | 0.42166 | 0.32 |
| G078 | 138.8 | 167 | 0.71073 | 0.54 |
| G086 | 100.7 | 150 | 0.82027 | 0.45 |
| G088 | 110.7 | 155 | 0.77072 | 0.32 |
| G091 | 78 | 149 | -0.32186 | 0.35 |
| G092 | 78 | 159 | -1.34659 | 0.25 |
| G093 | 123.4 | 165 | 0.32159 | 0.36 |
| G095 | 90.7 | 150 | 0.30963 | 0.27 |
| G096 | 98 | 150 | 0.68757 | 0.57 |
| G097 | 117.9 | 151 | 1.48686 | 0.25 |
| G098 | 207.7 | 182 | 1.34586 | 0.15 |
| G099 | 109.3 | 155 | 0.70841 | 0.22 |
| G200 | 136.1 | 168 | 0.51981 | 0.35 |
| G203 | 136 | 173 | 0.05232 | 0.51 |
| G204 | 76 | 142 | 0.3039 | 0.21 |
| G206 | 80.7 | 152 | -0.46882 | 0.29 |
| G207 | 112.5 | 161 | 0.25364 | 0.28 |
| G208 | 134.3 | 171 | 0.17442 | 0.18 |
| G209 | 175.1 | 174 | 1.21006 | 0.23 |
| G210 | 146 | 173 | 0.40288 | 0.44 |
| G211 | 150.1 | 160 | 1.76708 | 0.12 |
| G217 | 208.6 | 171 | 2.34797 | 0.36 |
| G219 | 264.9 | 187 | 2.12608 | 0.33 |
| G225 | 93.4 | 145 | 0.9814 | 0.31 |
| G226 | 104.3 | 145 | 1.51881 | 0.16 |
| G227 | 199.6 | 176 | 1.67768 | 0.20 |
| G229 | 79.8 | 142 | 0.54104 | 0.37 |
| G236 | 146 | 165 | 1.14923 | 0.16 |
| G237 | 87 | 139 | 1.29175 | 0.19 |

**Table S6.** The first three principal component (PC) axes of habitat use in grizzly bears (*Ursus arctos*) in Alberta, Canada. The percent variance explained by each axis is included in parentheses and descriptions of each habitat variable are in supplemental Table 5.

|  |  |  |  |
| --- | --- | --- | --- |
| Habitat variable | PC1 (49%) | PC2 (23%) | PC3 (13%) |
| wettree | 0.34 | -0.04 | 0.51 |
| regencut | 0.70 | -0.47 | -0.37 |
| shrub | -0.38 | -0.07 | -0.58 |
| wetherb | 0.14 | -0.10 | 0.31 |
| upherb | -0.36 | -0.31 | 0.21 |
| nonveg | -0.29 | -0.28 | 0.36 |
| upforest | 0.18 | 0.77 | -0.00 |

**Table S7.** Model results examining variation in mass, total length, and body condition. Bold indicates 95% credible intervals that did not overlap 0. For each model, *x* denotes and interaction term between sex and homozygosity, where *y* allowed homozygosity to vary by management unit. PC1/2 is in reference to habitat use scores from the principal components analysis. Clusters (*k*) were categorical variables based on each individuals highest assignment: the genetic *k* was 3, so the two values reported are relative to the third cluster. Model number is in reference to Supplemental Table S2.

| Model No. | DIC | Sex | Age | Northing | Spring capture | Summer capture | % Hom | % Hom 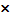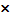 Sex | Genetics | | Habitat | | Overall intercept (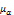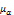) | Overall homozygosity  (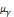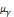) |
| --- | --- | --- | --- | --- | --- | --- | --- | --- | --- | --- | --- | --- | --- | --- |
|  |  |  |  |  |  |  |  |  | *K*1/2 | PC1/2 | *K* | PC1/2 |  |  |
| Body condition |  |  |  |  |  |  |  |  |  |  |  |  |  |  |
| 1 | 163.5 | **0.97** | **0.07** | 0.16 | 0.49 | 0.68 | - | - | - | - | - | - | -0.68 | - |
| 2 | 165.1 | **0.97** | **0.07** | 0.17 | 0.45 | 0.66 | 0.63 | - | - | - | - | - | -0.87 | - |
| 2^x^ | 167.2 | 1.14 | **0.08** | 0.17 | 0.44 | 0.64 | 0.81 | -0.54 | - | - | - | - | -0.92 |  |
| 2^y^ | 163.7 | **0.97** | **0.08** | 0.34 | 0.46 | 0.60 | - | - | - | - | - | - | -0.86 | 0.64 |
| 3 | 161.5 | **0.68** | **0.07** | -0.12 | 0.41 | 0.6 | 0.78 | - | -0.22/-0.42 | - | **0.71** | - | **-1.65** | - |
| 4 | 149.7 | **0.7** | **0.07** | -0.12 | 0.41 | 0.46 | 0.81 | - | - | -0.21/**0.36** |  | **1.48**/0.27 | -0.76 | - |
| Mass |  |  |  |  |  |  |  |  |  |  |  |  |  | - |
| 1 | 31.99 | **0.55** | **0.05** | 0.09 | 0.09 | 0.08 | - | - | - | - | - | - | **4.11** | - |
| 2 | 29.33 | **0.55** | **0.05** | 0.1 | 0.05 | 0.07 | **0.63** | - | - | - | - | - | **3.92** | - |
| 2^x^ | 29.56 | **0.82** | **0.05** | 0.12 | 0.04 | 0.04 | **0.9** | -0.83 | - | - | - | - | **3.84** | - |
| 2^y^ | 29.73 | **0.55** | **0.05** | 0.13 | 0.06 | 0.06 | - | - | - | - | - | - | **3.89** | 0.68 |
| 3 | 24.1 | **0.45** | **0.05** | -0.6 | 0.01 | 0.005 | **0.65** | - | -0.06/-0.07 | - | **0.29** | - | **3.56** | - |
| 4 | 24.6 | **0.46** | **0.05** | -0.03 | 0.02 | -0.04 | 0.56 | - | - | -0.04/0.01 | - | **0.45**/0.3 |  | - |
| Length |  |  |  |  |  |  |  |  |  |  |  |  |  | - |
| 1 | -145.4 | **0.11** | **0.01** | -0.01 | -0.002 | -0.02 | - | - | - | - | - | - | **4.94** | - |
| 2 | -148.1 | **0.11** | **0.01** | -0.01 | -0.01 | -0.02 | **0.15** | - | - | - | - | - | **4.89** | - |
| 2^x^ | -147.6 | **0.17** | **0.1** | -0.002 | -0.2 | -0.03 | **0.22** | -0.21 | - | - | - | - | **4.87** | - |
| 2^y^ | -140.9 | **0.11** | **0.01** | -0.01 | -0.01 | -0.01 | - | - | - | - | - | - | **4.88** | 0.19 |
| 3 | -146.2 | **0.09** | **0.01** | -0.04 | -0.02 | -0.04 | **0.15** | - | -0.01/0.0003 | - | **0.05** | - | **4.82** | - |
| 4 | -144.4 | **0.1** | **0.01** | -0.02 | -0.02 | -0.04 | 0.12 | - | - | 0.003/-0.2 | - | 0.05/0.07 | **4.92** | - |
