# Supplementary material for: Linking genotype, ecotype, and phenotype in an intensively managed large carnivore
Source: Evol Appl. 2013 Dec 4;7(2):301–12. doi: 10.1111/eva.12122 (PMC3927890; doi:10.1111/eva.12122)
Supplement: Supplementary file 6 [file eva0007-0301-sd6.docx]

Figure S1. Map showing individual PC1-3 scores of grizzly bears (*Ursus* arctos) in Alberta, Canada: a-c) based on genotypic data; d-f) based on habitat-use data. Six population units are identified: 1) Castle, 2) Livingstone, 3) Clearwater, 4) Yellowhead, 5) Grande Cache, and 6) Swan Hills.

Figure S2. Map showing individual PC1-3 scores of grizzly bears (*Ursus* arctos) in Alberta, Canada: a-c) based on habitat-use data. Six population units are identified: 1) Castle, 2) Livingstone, 3) Clearwater, 4) Yellowhead, 5) Grande Cache, and 6) Swan Hills.

Figure S3. Plots showing the relationship between a) coancestry and geographic distance; b) relatedness (QG) and geographic distance; c) coancestry and ecological distance (PC2), and d) relatedness and ecological distance (PC1). Table 1 shows the Pearson correlation and significance of the relationship.
